# Focal amyloid and asymmetric tau in an imaging-to-autopsy case of clinical primary progressive aphasia with Alzheimer disease neuropathology

**DOI:** 10.1186/s40478-022-01412-w

**Published:** 2022-08-09

**Authors:** Adam Martersteck, Ivan Ayala, Daniel T. Ohm, Callen Spencer, Christina Coventry, Sandra Weintraub, Eileen H. Bigio, M. -Marsel Mesulam, Changiz Geula, Emily Rogalski

**Affiliations:** 1 Mesulam Center for Cognitive Neurology and Alzheimer’s Disease, Northwestern University (NU) Feinberg School of Medicine, 300 E. Superior St., Tarry 8, Chicago, IL 60611 USA; 2 Department of Radiology, NU Feinberg School of Medicine, Chicago, IL USA; 3 Department of Neurology, University of Pennsylvania Perelman School of Medicine, Philadelphia, PA USA; 4 Department of Psychiatry and Behavioral Sciences, NU Feinberg School of Medicine, Chicago, IL USA; 5 Department of Pathology, NU Feinberg School of Medicine, Chicago, IL USA; 6 Department of Neurology, NU Feinberg School of Medicine, Chicago, IL USA; 7 Department of Cell and Developmental Biology, NU Feinberg School of Medicine, Chicago, IL USA

**Keywords:** Alzheimer disease, Primary progressive aphasia, Amyloid, Tau, Positron emission tomography, Stereology, Florbetapir, Flortaucipir

## Abstract

Quantification of in vivo amyloid and tau PET imaging relationships with postmortem measurements are critical for validating the sensitivity and specificity imaging biomarkers across clinical phenotypes with Alzheimer disease neuropathologic change (ADNC). This study examined the quantitative relationship between regional binding of in vivo ^18^F-florbetapir amyloid PET and ^18^F-flortaucipir tau PET with postmortem stereological counts of amyloid plaques and neurofibrillary tangles (NFT) in a case of primary progressive aphasia (PPA) with ADNC, where neurodegeneration asymmetrically targets the left hemisphere. Beginning 2 years prior to death, a 63-year-old right-handed man presenting with agrammatic variant PPA underwent a florbetapir and flortaucpir PET scan, and neuropsychological assessments and magnetic resonance imaging sessions every 6 months. Florbetapir and flortaucpir PET standard uptake value ratios (SUVRs) were quantified from 8 left and right hemisphere brain regions with stereological quantification of amyloid plaques and NFTs from corresponding postmortem sections. Pearson’s correlations and measures of asymmetry were used to examine relationships between imaging and autopsy measurements. The three visits prior to death revealed decline of language measures, with marked progression of atrophy. Florbetapir PET presented with an atypical focal pattern of uptake and showed a significant positive correlation with postmortem amyloid plaque density across the 8 regions (*r* = 0.92; *p* = 0.001). Flortaucipir PET had a left-lateralized distribution and showed a significant positive correlation with NFT density (*r* = 0.78; *p* = 0.023). Flortaucipir PET and NFT density indicated a medial temporal lobe sparing presentation of ADNC, demonstrating that AD does not always target the medial temporal lobe. This study adds additional evidence, in a non-amnestic phenotype of ADNC, that there is a strong correlation between AD PET biomarkers, florbetapir and flortaucipir, with quantitative neuropathology. The atypical and focal presentation of plaque density and florbetapir PET uptake suggests not all amyloid pathology presents as diffuse across neocortex.

## Introduction

The two pathologic hallmarks of Alzheimer disease (AD) neuropathologic change (ADNC) are extracellular aggregation of β-amyloid (Aβ) plaques and intraneuronal neurofibrillary tangles (NFTs) [19, 40]. In the last decade, technology to detect these pathologies in vivo has rapidly accelerated. ^18^F-florbetapir was among the first fluorinated amyloid tracers to be developed for research and clinical use [5, 74], approved by the Food and Drug Administration (FDA) as Amyvid™ in 2012 to indicate moderate or frequent density of Aβ plaques. ^18^F-flortaucipir (formerly AV1451 and T807) was subsequently developed, has been found to selectively bind to AD-type paired helical filament tau by autoradiography [29, 32, 58, 76], and has recently been approved by the FDA (Tauvid™, 2020).

The initial imaging-to-autopsy studies validating positron emission tomography (PET) tracers primarily included participants with a ‘typical’ amnestic presentation in late stages of disease, selected based on a projected life expectancy of less than 6 months [7, 8, 13, 56, 57]. The phase 3 and follow-up studies determined the efficacy of each tracer by comparing a visual PET read classification (e.g., positive vs. negative) and semiquantitative neuropathologic score or stage (e.g., CERAD score; Braak stage IV and below vs. Braak V/VI) [6–8, 12, 13, 15, 20, 30, 56, 57, 65, 73]. But there have been few quantitative studies in AD examining the association between florbetapir or flortaucipir PET standard uptake value ratios (SUVRs) with either biochemical assays [3, 48], immunostained stereological counting or percent area occupied measures [27, 30, 52, 64, 75]. Quantitative studies are key to determining the sensitivity and specificity of in vivo biomarkers and further validation is required for amnestic and non-amnestic AD.

The present study extends the current research by providing regional quantification of PET and neuropathologic measures of amyloid and tau in a non-amnestic presentation of AD. Specifically, it is based on the postmortem stereological quantitation of plaques and tangles in multiple brain regions of a PPA participant that came to autopsy with ADNC and had received florbetapir and flortaucipir PET scans close to death. We hypothesized that florbetapir PET would have a strong relationship with postmortem measured Aβ plaques (APs), and that flortaucpir PET would have a strong relationship with postmortem measured NFTs across the sampled brain regions.

## Methods

### Participant evaluation

The participant was recruited into Northwestern’s PPA Research Program after receiving a clinical diagnosis of PPA, made by a behavioral neurologist (M.-M.M.), based on the presence of an initially isolated and progressive language impairment consistent with a neurodegenerative etiology [16, 36, 37]. Participation included magnetic resonance imaging (MRI) and neuropsychological testing at 6-month intervals, and a ^18^F-florbetapir PET and a ^18^F-flortaucipir PET scan.

Neuropsychological evaluation included tests that measured global aphasia severity, repetition, object naming, grammar, word comprehension, sentence comprehension, and word fluency. The Western Aphasia Battery (WAB) [25] aphasia quotient (WAB-AQ), a measure of aphasia severity, is a composite calculated from tests of naming, repetition, auditory comprehension, and spontaneous speech. The 6 most difficult items (items 10–15) from the WAB repetition subset were used to grade repetition. Naming was assessed with the 60-item version of the Boston Naming Test (BNT) [24]. Auditory lexical-semantic processing was assessed with a subset of moderately difficult items (items 157–192) from the fourth edition of the Peabody Picture Vocabulary Test (PPVT) [11]. Grammatical processing was judged on a composite score from 15 noncanonical sentences from the Northwestern Anagram Test [71] and 15 noncanonical sentences from the Sentence Production Priming Test of the Northwestern Assessment of Verbs and Sentences. Handedness was determined by the Edinburgh Inventory [45]. See [16, 37] for further details on the quantitative thresholds used for the diagnosis and subtyping.

Northwestern’s Institutional Review Board approved the study and written informed consent was obtained.

### MRI and PET image acquisition and processing

At each visit, the participant underwent an MRI on a 3 T Siemens TIM Trio (Munich, Germany). A T_1_-weighted 3D magnetization-prepared rapid gradient echo (MPARGE; TR = 2300 ms, TE = 2.91 ms, TI = 900 ms, flip angle = 9°, FoV = 256 mm) recorded 176 slices with a slice thickness of 1.0 mm and in-plane resolution of 1.0 × 1.0mm^2^. Thirty-five normal controls (NC), previously described [53], were used to calculate z-score cortical thickness maps. On average, the NC group was 62.4 (± 7.0) years old with 16.0 (± 2.4) years of education. Seventeen out of 35 were male. All were right-handed. The PPA participant and NC participants were scanned on the same MRI with the same T_1_-weighted sequence described above.

The PPA participant completed florbetapir PET and flortaucipir PET imaging at the second and third visits, respectively, on a Siemens Biograph TruePoint/TrueV PET-CT system. A bolus intravenous injection of 10.5 mCi ^18^F-florbetapir or 10.0 mCi ^18^F-flortaucipir was used. A low-dose CT and 20 min of PET scanning in dynamic list-mode were acquired 50 min (florbetapir) or 85 min (flortaucipir) post-injection. Data were reconstructed into 5-min frames with the Alzheimer Disease Neuroimaging Initiative 3 protocol, with an iterative ordered subset expectation–maximization algorithm with attenuation correction on a 336 × 336 × 109 grid.

MRI and PET image processing were carried out as previously described [34]. Briefly, for the PPA and NC participants, the MPRAGE was processed with FreeSurfer v6.0.0 (surfer.nmr.mgh.harvard.edu) cross-sectional and longitudinal pipelines [51] to generate surfaces and calculate cortical thickness. Topological errors were iteratively corrected based on established guidelines [61].

For PET processing, the 4 × 50–70 min frames for florbetapir or the 3 × 85–100 min frames for flortaucipir were motion corrected and the mean image was rigidly registered to the unbiased longitudinal [50] native T_1_-space for calculation of the reference region and surface projection. For the florbetapir reference region, the Desikan-Killiany FreeSurfer-defined whole cerebellum was used. For the flortaucipir reference region, the previously described method [2] was used to generate a segmentation of the inferior cerebellar gray free of hotspot clusters. Briefly, the participant’s T_1_ was used to calculate the non-linear warp from longitudinal native space to the MNI-152 template with Statistical Parametric Mapping v12 (fil.ion.ucl.ac.uk/spm) DARTEL [1] to reverse warp the SUIT toolbox [10] cerebellar lobules to native space. The inferior cerebellar binary segmentation was smoothed and combined with the FreeSurfer-defined cerebellar grey and contiguous hotspot voxels were removed from the reference region mask.

The PET data were analyzed with and without partial volume correction (PVC). The modified Müller-Gärtner PVC from FreeSurfer [17, 18] was used as described previously [34]. The approximate point spread function was set at 4.5mm^3^, based on prior studies of the Biograph TruePoint/TrueV [22, 26]. Corrected and uncorrected voxel intensities were projected to the corresponding left and right hemisphere surface vertices from the voxel intensity along the surface normal (i.e., the line orthogonal to the pial and white surfaces, along the vector that describes cortical thickness).

All pre-processing was kept in the unbiased longitudinal native space. The only surfaces spherically warped to a common space (fsaverage) were the thickness maps, which required the PPA participant and NC data to be in the same stereotaxic coordinate space to calculate z-scores. The z-score maps were computed at each vertex as $\frac{\mathrm{x }-\upmu }{\upsigma }$, where x is the PPA participant thickness, μ and σ the mean and standard deviation of the NC group thickness, respectively.

PET and MR images were thoroughly inspected at each processing stage for artifacts or errors that may have led to focality or lateralization effects (e.g., improper registration of the PET-to-CT for attenuation correction, misalignment of the MRI-to-PET, surface distortions, tissue class segmentation for PVC).

### Neuropathologic diagnosis

Standard processing methods for determining the neuropathologic diagnosis were performed [19, 39, 40]. Briefly, the brain was examined for gross and microscopic pathology. Tissue blocks were paraffin-embedded, cut at 5 μm thickness, mounted on charged glass slides, and deparaffinized in xylenes. Standard staining protocols were employed, including hematoxylin and eosin, thioflavin-S, and Gallyas. Immunohistochemical preparations included phosphorylated tau (AT8 and PHF-1), amyloid-β (4G8), p62, phosphorylated TDP-43, and α-synuclein. A neuropathologist (E.H.B.) examined tissue across 37 regions and made a semiquantitative rating of none, mild, moderate, or severe for each possible pathology.

### Unbiased stereology and imaging quantification

Eight regions of interest (ROI) were selected based on the variability in cortical PET binding and left–right asymmetry, with the goal to sample from a wide range of PET uptake and neuropathology. The resultant ROIs selected were the left and right middle frontal, left and right entorhinal, left and right superior temporal, and left and right middle temporal cortex. For correspondence between the postmortem tissue ROIs and PET imaging ROIs, we followed the previously described protocol for tissue-to-MRI registration [43, 44]. Briefly, tissue blocks that contained each region were manually co-registered to the T_1_-weighted MRI by an expert neuroanatomist (C. G.). After the glass slides were marked to indicate the extent of stereological counting along the cortex, the imaging ROIs were created voxel-wise by filling the corresponding cortical ribbon along the vertical axis the tissue was cut at. Limited by the spatial resolution of the in vivo imaging, the imaging ROI that best matched each coronal-cut tissue slide was a vertical stripe, 3 mm thick in the x-axis, and completely filling the cortical ribbon in the y- and z-axis.

The number of NFTs and amyloid plaques immunoreactive to PHF-1 and thioflavin-S antibodies were quantified using unbiased stereological methods, employing the optical fractionator probe of the StereoInvestigator Software (MBF Biosciences, MicroBrightfield Inc., Williston, VT, USA). Counting was done by an individual (I.A.) blinded to florbetapir PET and flortaucipir PET status and spatial uptake. Only dense-cored and neuritic APs were counted with thioflavin-S.

ROI were traced at 5× and counted at 63× across 5–6 serial sections. The number of counting sites was determined based on requirements for unbiased stereological quantification [59]. Briefly, the optical fractionator probe estimated the populations of each marker from all sections per ROI, with grid dimensions that varied to produce a coefficient of error ≤ 0.1.

For each post-mortem ROI, density of pathology was calculated by taking the estimated population and dividing by the planimetry volume to produce counts of NFTs or APs per mm^3^. For the PET imaging ROIs, we extracted the average tau PET or amyloid PET SUVR, with and without PVC, across the same in-register PET-to-autopsy ROIs.

### Statistics

Correlations between ROI-wise flortaucipir PET SUVR and PHF-1 NFT density or between florbetapir PET SUVR and thioflavin-S AP density were conducted with Pearson’s correlations using Python v3.6 and statsmodels package v0.9.0.

To examine the hemispheric differences in amyloid and tau, a within-modality by ROI laterality ratio between left hemisphere (LH) and right hemisphere (RH) density or SUVR was calculated as:

Laterality Index (LI_*ROI*_) = $\frac{{\mathrm{LH}}_{measure} -\mathrm{ R}{\mathrm{H}}_{measure}}{{\mathrm{average}}_{({\mathrm{LH}}_{measure}\mathrm{ , R}{\mathrm{H}}_{measure})}} *100$. The LI_*ROI*_ is the symmetrized percentage a region is lateralized compared to the average burden between left and right regions. Positive values indicate greater leftward asymmetry and negative values greater rightward asymmetry. For example, in hypothetical region X, a LI_x_ of + 30 with PHF1 density indicates LH region X has 30% more tau burden than RH region X, relative to the average density of tau pathology in LH region X and RH region X.

To better characterize this participant among AD pathologic subtypes [41], we calculated a within-modality ratio of tau burden, a neocortical-to-entorhinal index. The neocortical-to-entorhinal index was calculated as: $\frac{{\overline{\mathrm{x}} }_{\mathrm{neocortical}} - {\overline{\mathrm{x}} }_{\mathrm{entorhinal}}}{{\mathrm{average}}_{({\overline{\mathrm{x}} }_{\mathrm{neocortical}}\mathrm{ , }{\overline{\mathrm{x}} }_{\mathrm{entorhinal}})}} *100$. Where x̅_neocortical_ is the average flortaucipir PET SUVR or PHF-1 NFT density of the six ROIs from the bilateral superior temporal, middle temporal, and middle frontal regions, and x̅_entorhinal_ is the average of the LH and RH entorhinal regions. The neocortical-to-entorhinal index is a symmetrized percentage that indicates the percent burden of tau pathology in lateral cortical regions compared to entorhinal regions, relative to the average burden across neocortical ROIs and entorhinal ROIs, with positive numbers indicating greater lateral cortical burden and negative numbers greater entorhinal burden. For example, a neocortical-to-entorhinal index of + 20 indicates 20% higher tau burden in the six lateral cortical ROIs compared to the entorhinal ROIs, relative to the average burden between neocortical and entorhinal ROIs.

## Results

### Participant characteristics: demographics, clinical history, and atrophy measures

At the initial visit, the participant was 63 years old, with a 7-year history of progressive language difficulties consistent with the agrammatic variant of PPA. He was a right-handed (+ 80 Edinburgh Handedness Inventory [45]) Caucasian male with 20 years of education. At the initial visit, he showed agrammatism in language production, impaired comprehension of syntactically complex non-canonical sentences, motor speech impairments, spared single word comprehension, and spared object knowledge (Table 1, baseline). The lower scores on the BNT can partially be explained by the mild apraxia of speech. At the initial neurological exam, the participant had intact mental calculations. At each visit, he was able to copy line drawings from the Three Words Three Shapes (3W3S) test [70, 72] without error. Further, on the delayed recall memory test of the 3W3S, the participant was able to correctly identify the three line drawings. On a survey of early-life learning problems, he reported difficulty with reading as a child. Apolipoprotein E genotyping was ε3/ε4.

**Table 1:** Neuropsychological performance and functional decline over time

Visit	WAB-AQ (100)	WAB-Rep (66)	BNT (60)	PPVT (36)	NAT-nc (15)	Rivermead (10)	CDR-global (*)	CDR-language (*)	ADLQ (100)	NPI-Q severity (36)
Baseline	68.30	23	39	35	2	10	0.5	2	34.85	1
6-month	54.50	19	25	27	4	10	0.5	2	42.11	3
12-month	39.80	5	22	32	N/A	8	0.5	2	46.67	6
18-month	35.10	1	0	21	0	8	1	3	54.67	7

Neuropsychological performance and functional questionnaire at each visit. WAB = Western Aphasia Battery; WAB-AQ = WAB aphasia quotient; WAB-Rep = WAB repetition subtest; BNT = Boston Naming Test; PPVT = Peabody Picture Vocabulary Test; NAT-nc = Sentence production scores on a composite of 15 noncanonical sentences from the Northwestern Anagram Test; Rivermead = Rivermead Behavioural Memory Test; CDR = Clinical Dementia Rating; ADLQ = Activities of Daily Living Questionnaire; NPI-Q severity = Neuropsychiatric Inventory-Questionnaire, sum of severity scores; N/A = not available. Higher numbers indicate better performance for the WAB-AQ, WAB-Rep, BNT, PPVT, NAT-nc, and Rivermead. * The CDR is an ordinal scale with possible values of 0, 0.5, 1, 2, or 3. Higher numbers the CDR-global (based on six cognitive domains) and CDR-language (language domain only) indicate increased impairment. Higher numbers on the ADLQ and NPI-Q indicate increased functional impairment

**Fig. 1:**
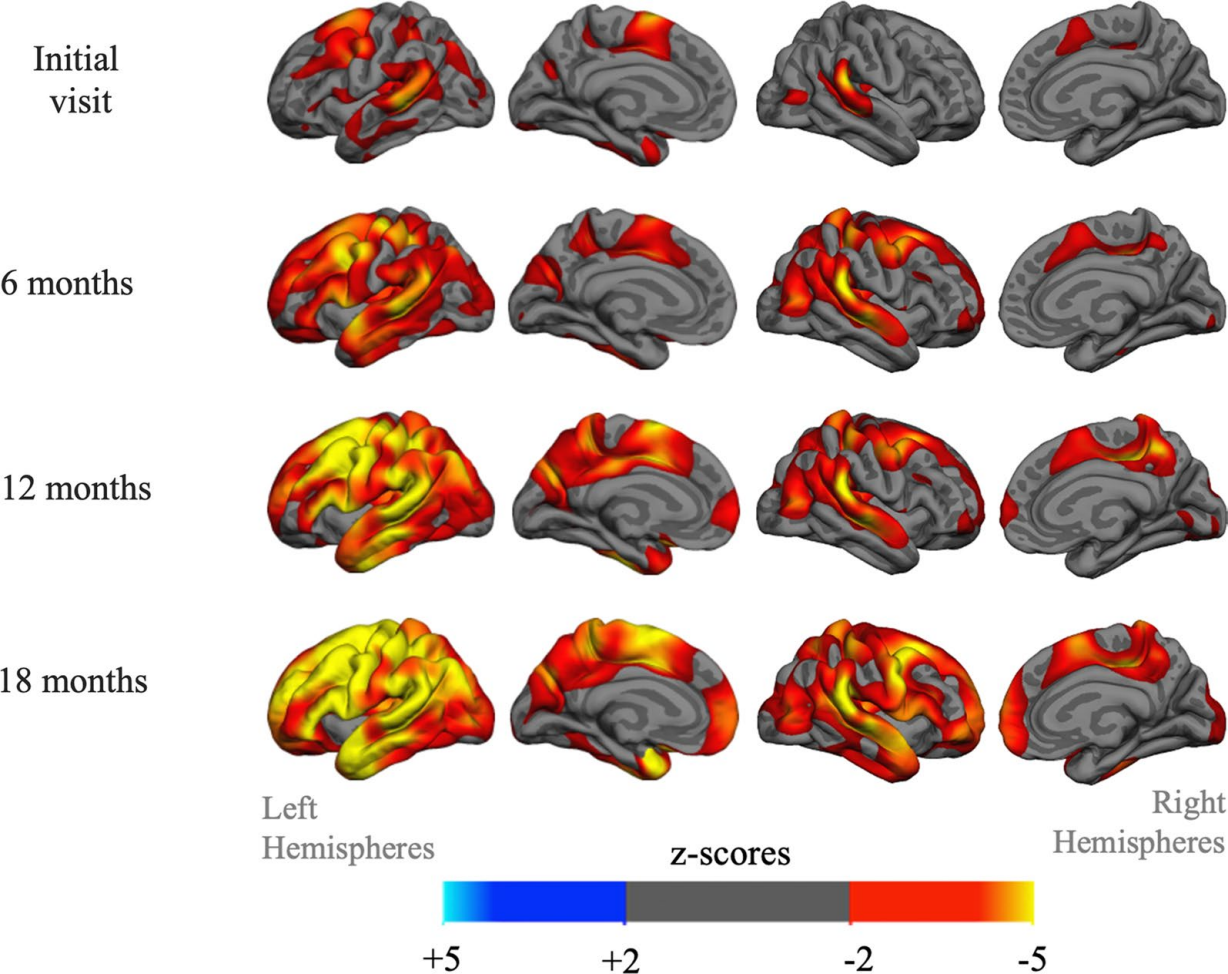
Progression of atrophy. Structural T_1_-weighted MRI derived cortical thickness differences between the primary progressive aphasia (PPA) participant and 35 normal controls (NC). The z-score map displays vertices greater than 2 standard deviations from the mean of the NC group. Warmer colors indicate less cortical thickness for the PPA participant. Left perisylvian temporal cortex, middle frontal cortex, and supplementary motor regions are the most abnormal early in disease course. A rapid progression of cortical thinning is detectable at each subsequent visit, with greater involvement of left hemisphere regions

The participant completed 3 follow-up visits with marked decrease on measures of global aphasia, naming, repetition, measures of sentence production, and decreases in functional measures (Table 1). The FreeSurfer cortical thickness analysis estimated thinner cortex in the left-lateralized language network at baseline, with increasing atrophy across the brain at 6-month intervals (Fig. 1).

### Postmortem findings and diagnosis

The participant passed away 6.5 months after the final visit, 13 months after the flortaucipir PET scan, and 19 months after the florbetapir PET scan. The postmortem interval was 39 hours. Inspection of the brain revealed asymmetric temporal and parietal atrophy. There was minimal neuronal loss and gliosis in the hippocampus.

Thioflavin-S and Gallyas stains revealed abundant APs and NFTs in the neocortex. NFTs, and both neuritic and diffuse plaques were rated as severe with noticeable left greater than right lateralization in most regions. Overall, plaques were consistent with Thal phase 5, NFTs with Braak stage VI, and neuritic plaques were scored as frequent, with a final diagnosis of high ADNC, “A3, B3, C3”.

There were no contributing co-pathologies found. There were no signs of Lewy bodies, p62 positive pathology, or TDP-43 immunostaining. Mild to moderate non-occlusive cerebrovascular disease was found, due to mild arteriosclerosis and mild to moderate subcortical white matter rarefaction. There were no large infarcts or hemorrhages. Cerebral amyloid angiopathy (CAA) was rated as absent in 10 of 12 regions and rated mild in the right middle frontal and left middle temporal regions. Previous studies have shown that CAA burden in AD does not significantly contribute to cortical amyloid PET uptake [20, 35].

### Characterization of florbetapir amyloid PET and postmortem measured amyloid plaque density

**Fig. 2.**
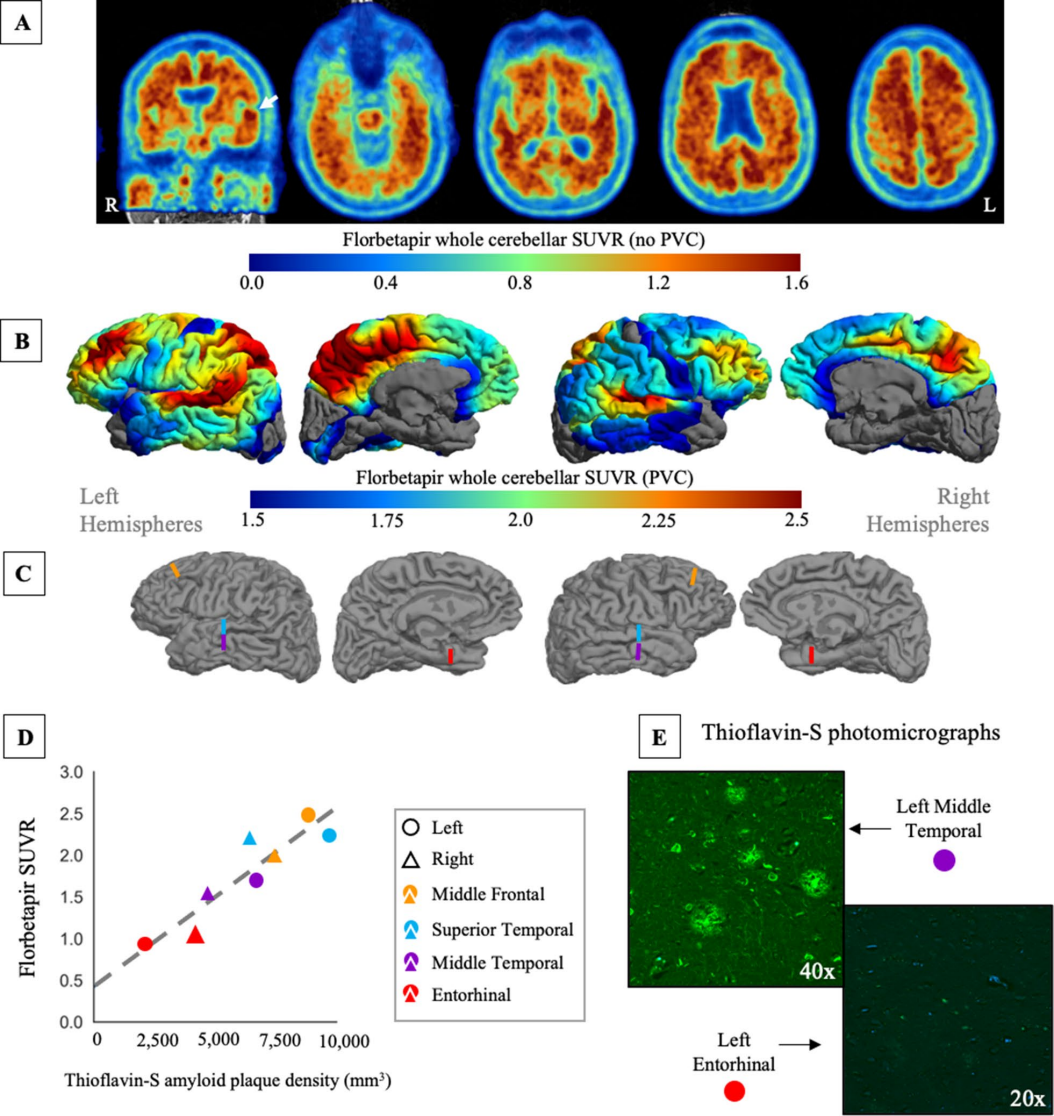
^18^F-florbetapir amyloid PET and thioflavin-S amyloid plaque density show close correspondence. **A** The florbetapir PET standard uptake value ratio (SUVR) image, acquired 19 months before death, referenced by the whole cerebellum and without partial volume correction (PVC), overlaid on the participants MRI in native space. A coronal and four descending axial slices are displayed. The white arrow points towards the focal binding in the left superior temporal region. **B** The same PET data as in A with volume-to-MRI-surface projection and PVC applied. The focality and asymmetry of uptake were pronounced, specifically in the left middle frontal, superior temporal, and precuneus regions. **C** The eight color bars on the native MRI surface reconstruction indicate where postmortem dissections and stereological counting was performed. **D** There was a strong correlation between thioflavin-S amyloid plaque density and florbetapir PET SUVR across the eight regions (*r* = 0.92, *p* = 0.0013). (E) Photomicrographs from the fluorescent thioflavin-S staining, from the middle temporal region and entorhinal region, at 40× and 20× magnification

Visual inspection of the ^18^F-florbetapir amyloid PET scan and surface projection revealed a focal pattern of uptake along the superior temporal and middle frontal gyri, and precuneus (Fig. 2A, B). The regional PET SUVR measurements, stereological densities, and laterality indices are presented in Table 2. Thioflavin-S plaque density from the unbiased stereology had a wide range of AP burden across the eight ROIs (see Fig. 2C for visualization of ROIs). Density ranged from the lowest in the left entorhinal (2,087 AP/mm^3^) to highest in the left middle frontal (8,784 AP/mm^3^). Florbetapir tracer uptake and AP density measurements were left-lateralized in all regions except for the entorhinal cortex (Table 2). Notably, across all ROIs, the post-mortem AP density measurements had higher laterality indices compared to PET ROIs.

**Table 2:** PET SUVR and stereological density per region of interest and corresponding laterality index and neocortical-to-entorhinal index

ROI	Hemisphere/Laterality	Florbetapir PVC SUVR	Thioflavin-S AP density mm^3^	Flortaucipir PVC SUVR	PHF-1 NFT density mm^3^
Entorhinal	L	0.95	2,086	1.29	3,745
	R	1.02	4,264	1.18	2,640
	Laterality index	− 7	− 69	+ 9	+ 35
Middle Frontal	L	2.48	8,784	2.99	50,668
	R	1.98	7,386	1.87	28,474
	Laterality index	+ 22	+ 17	+ 46	+ 56
Superior Temporal	L	2.23	9,005	3.00	37,741
	R	2.19	6,375	2.88	17,540
	Laterality index	+ 2	+ 34	+ 4	+ 73
Middle Temporal	L	1.69	6643	2.60	18,320
	R	1.54	4634	1.81	11,952
	Laterality index	+ 9	+ 36	+ 36	+ 42
Neocortical-to-entorhinal index		Not computed	Not computed	+ 68	+ 158

The left (L) and right (R) hemisphere stereological density estimations and PET measurements by region of interest (ROI). PET measures have been partial volume corrected (PVC) and referenced by whole cerebellum (florbetapir) or inferior cerebellar grey (flortaucipir) to create a standard uptake value ratio (SUVR). Stereological counts are either amyloid plaque (AP) or neurofibrillary tangle (NFT) densities. The calculation for the laterality index and neocortical-to-entorhinal index are outlined in the Methods. Briefly, a positive laterality index indicates L > R uptake or density, while a positive neocortical-to-entorhinal index indicates greater uptake or density in the six lateral cortical ROIs compared to the two entorhinal ROIs

The correlational analysis showed a strong relationship between the PVC florbetapir SUVR and thioflavin-S AP density in the eight ROIs (*r* = 0.92, *p* = 0.0013; Fig. 2D). The relationship was similar without PVC (*r* = 0.85, *p* = 0.0081). Photomicrographs of fluorescent thioflavin-S immunostaining demonstrate the AP burden (Fig. 2E).

### Characterization of flortaucipir tau PET and postmortem measured neurofibrillary tangle density

**Fig. 3.**
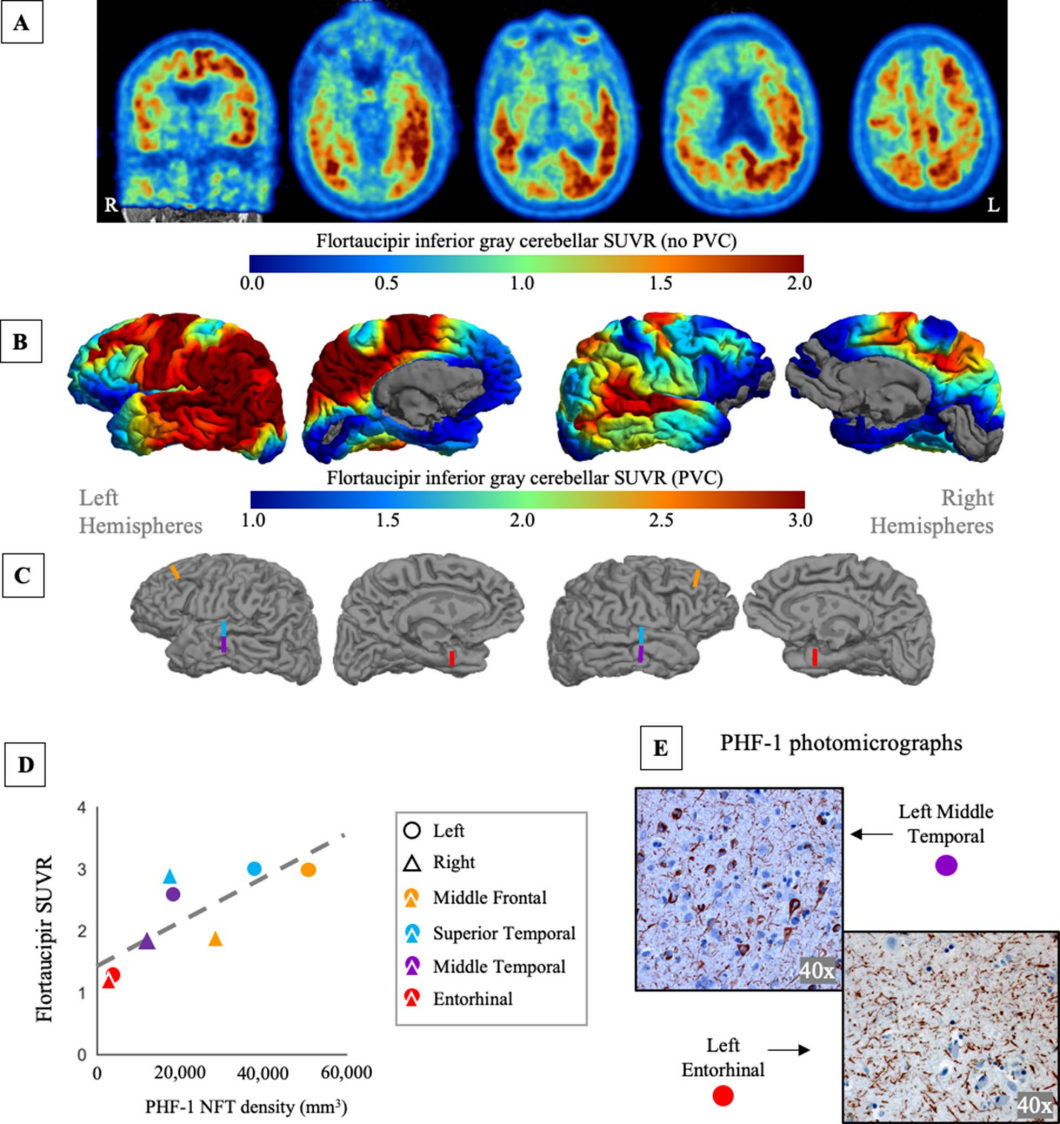
^18^F-flortaucipir tau PET and PHF-1 neurofibrillary tangle density visualization and association. **A** The flortaucipir PET standard uptake value ratio (SUVR) image, acquired 13 months before death, referenced by inferior cerebellar grey and without partial volume correction (PVC), overlaid on the participants MRI in native space. A coronal and four descending axial slices are displayed. **B** The same PET data as in A with volume-to-MRI-surface projection and PVC applied. The uptake is highly left-lateralized, favoring the left hemisphere perisylvian language network. **C** The eight color bars on the native MRI surface reconstruction indicate where postmortem dissections and stereological counting was performed. **D** There was a strong correlation between PHF-1 neurofibrillary tangle (NFT) density and flortaucipir PET SUVR across the eight regions (*r* = 0.78, *p* = 0.023). **E** Photomicrographs from the PHF-1 immunostaining to visualize NFTs, counter-stained by cresyl violet to visualize neuronal cell (Nissl) bodies, from the middle temporal and entorhinal regions, at 40× magnification

The ^18^F-flortaucipir PET scan had a less focal presentation and a more typical PPA pattern, resembling previous cross-sectional and longitudinal studies of flortaucipir PET [34] and cortical atrophy [54] (Fig. 3A, B). As expected, the atrophy pattern (Fig. 1) closely resembles the flortaucipir PET surface projection. Off-target flortaucipir binding to the choroid plexus and extra-cortical hotspots [2] were absent.

PHF-1 NFT counts from the unbiased stereology ranged from 2640 NFT/mm^3^ in the right entorhinal cortex to 50,668 NFT/mm^3^ in the left middle frontal ROI. Both entorhinal cortices were relatively spared of tau pathology. The neocortical-to-entorhinal index was + 158, indicating 158% higher tau burden in the 6 lateral hemisphere ROIs (average 27,449 NFT/mm^3^) compared to the entorhinal regions (average 3193 NFT/mm^3^), relative to the average burden across regions. The PVC flortaucipir PET had a similarly skewed pattern, with a neocortical-to-entorhinal index of + 68. In the context of neuropathologically defined subtypes of AD [41], this would be a medial temporal lobe sparing presentation.

There was a significant relationship between PVC flortaucipir SUVR and PHF-1 NFT density (*r* = 0.78, *p* = 0.023; Fig. 3D). The relationship was not significant when analyzed without PVC (*r* = 0.57, *p* = 0.1414). PET imaging measures and NFT density measurements had a high degree of laterality (Table 2). NFT density measurements were more left-lateralized (LI_all-region_ average =  + 51, range + 35 to + 73) compared to PVC flortaucipir PET laterality (LI_all-region_ average =  + 24, range + 4 to + 46).

The largest disagreement between the different modalities’ laterality was when non-PVC flortaucipir data were used (LI_all-region_ average = 13, range − 6 to 31,). For example, in the superior temporal gyrus (STG), one of the most atrophic regions, PHF-1 density LI_STG_ =  + 73 and non-PVC flortaucipir LI_STG_ = − 6. This indicates minor right-lateralized asymmetry for the PET signal, while the stereological counting had strong leftward asymmetry. Across all ROIs, PVC decreased this mismatch, and leftward asymmetry of PET signal was more in line with stereological density compared to without PVC.

## Discussion

This study examined the longitudinal atrophy, molecular imaging, and neuropathologic features of a participant with PPA, who came to autopsy with high ADNC. The key findings were that the density of APs, measured by thioflavin-S, showed a strong correlation with the ^18^F-florbetapir PET scan 19 months before death. Further, the density of PHF-1 stained NFTs was correlated with the regional cortical uptake of the flortaucipir PET scan 13 months before death. These results further validate the two PET tracers for assessing underlying AD neuropathologic change.

Cortical florbetapir amyloid PET uptake was asymmetric and presented in a highly focal pattern, in left hemisphere regions. Previous studies of PPA-ADNC have reported mixed findings for hemispheric lateralization of amyloid PET [28, 33, 42, 49]. The focal pattern is less well described compared to typical ADNC Thal phases [69], which represents amyloid as diffuse and uniform across neocortex with early sparing of somatomotor regions.

Most studies of florbetapir have been efficacy studies examining the correspondence with visual reads, using semiquantitative Thal phases or neuritic plaque scores [6, 7, 68]. Clark and colleagues found 96% sensitivity and 100% specificity for the detection of moderate to frequent neuritic plaques [7]. Recent studies have found florbetapir visual reads to have accurate discrimination between Thal phases (above 0.85 receiver operating characteristic) [68]. There have been few quantitative studies of florbetapir and postmortem AP counts. Beach et al. [3] used postmortem ELISA to measure Aβ_40_ and Aβ_42_ in participants from the previous study [7], finding a strong relationship between global cortical florbetapir SUVR and Aβ_40_ (Spearman rho = 0.67) and Aβ_42_ (Spearman rho = 0.80). The current study, the first study to examine regional florbetapir SUVR alongside the density of APs by quantitative stereological counting, adds evidence that florbetapir is associated with dense-cored and neuritic amyloid plaque burden. Given the inherent uncertainty (i.e., smoothness) of PET technology, it is notable that the florbetapir PET captured the sharp gradient of amyloid plaques between left superior temporal (2.23 SUVR; 9,005 AP/mm^3^) and left middle temporal (1.69 SUVR; 6,643 AP/mm^3^). Importantly, we counted only neuritic plaques, which previous research [21] using fluorescent derivatives has shown to be the major contributor to amyloid PET signal (specifically for ^18^F-flutemetamol and ^11^C-Pittsburgh compound B). Further, the careful iterative surface reconstruction, lack of volume-wise smoothing, and PVC may have decreased potential sources of measurement error. In this case, PVC substantially improved our flortaucipir-to-NFT correlation, and our laterality scores were more in line with the postmortem counts than without PVC. Previous reports have shown PVC improves detection and longitudinal measurement in real and simulated PET data [4, 55, 66], with one study finding it increased variability for amyloid PET [60].

In contrast to the non-ADNC imaging-to-autopsy cases [9, 23, 31, 63], ADNC cases have shown high correspondence between flortaucipir and underlying tau pathology, backed by autoradiography studies [29, 32]. Lowe et al. [30] and Soleimani-Meigooni et al. [65] reviewed case series of non-ADNC and ADNC participants and found strong correlations between postmortem measures of AD-type tau and antemortem flortaucipir burden. Smith et al. [64] found significant correlations between antemortem flortaucipir and Gallyas and AT8 tau pathology in a PSEN1 mutation carrier. Their intrasomal tau correlation is in line with our flortaucpir and PHF-1 correlations. We chose to examine the relationship with PHF-1, which autoradiography had suggested has the best relationship with flortaucipir [29, 32]. Our better correlation for florbetapir PET with AP density (*r* = 0.92) compared to flortaucipir PET with NFT density (*r* = 0.78) may be explained by the relative change in pathology between PET scan and autopsy. It is possible that tau pathology accumulated faster, relative to APs, or advanced in NFT maturity stage, hypothesized to effect flortaucipir binding properties [29]. Additional studies are needed to examine longitudinal accumulation of tau pathology, and to further investigate the correlation between tau maturity and PET binding.

This participant presented with the agrammatic subtype of PPA, with motor speech impairments. This subtype is most commonly associated with a frontotemporal lobar degeneration tauopathy [38]. The AD pathology in this case reinforces that PPA subtypes have probabilistic relationships with underlying neuropathology [38]. The left hemisphere language network showed a vulnerability to tau pathology and atrophy consistent with our previous reports and others [14, 34, 46, 47, 54].

There are limitations to the present work. Foremost, this is a case report. It should not be interpreted that a certain density of pathology is equivalent to a specific SUVR from this data. This participant had a focal pattern of amyloid PET burden, which provided an ideal dynamic range across the cortex to investigate relationships. But the frequency of such focal burden in other AD cases is unclear as many studies examine data at the group level or across an aggregate set of cortical ROIs. We are encouraged by recent advancements in deep neural networks to recognize and quantify postmortem neuropathology [62, 67], as these advancements may allow for additional quantitative imaging-to-autopsy studies with more cases that sample from multiple brain regions.

## Conclusions

In this case study, our results provide further evidence of the strong relationship between molecular imaging of pathology with ^18^F-florbetapir for amyloid plaques and ^18^F-flortaucipir with AD-type tau pathology. Additionally, these results provide further evidence that molecular imaging of AD pathology in a non-amnestic variant has similar relationships as previously described in amnestic AD dementia.

## Data Availability

The data generated and analyzed during the current study are available, anonymized and de-faced, through our collaborative request process (brain.northwestern.edu).

## Abbreviations

AD: Alzheimer disease
ADNC: Alzheimer disease neuropathologic change
Aβ: β-Amyloid
NFT: Neurofibrillary tangle
PET: Positron emission tomography
SUVR: Standard uptake value ratio
PPA: Primary progressive aphasia
MRI: Magnetic resonance imaging
NC: Normal control
PVC: Partial volume correction
ROI: Region of interest
AP: β-Amyloid plaque
PHF: Paired helical filament
LH: Left hemisphere
RH: Right hemisphere
CAA: Cerebral amyloid angiopathy
